# Management of a supernumerary tooth fused to the labial surface of a permanent maxillary central incisor

**DOI:** 10.1002/ccr3.8179

**Published:** 2023-11-22

**Authors:** Hadi Assadian, Behnam Bolhari, Mehrfam Khoshkhounejad, Nazanin Chitsaz, Maryam Babaahmadi

**Affiliations:** ^1^ Department of Endodontics, School of Dentistry Tehran University of Medical Sciences Tehran Iran

**Keywords:** fused teeth, root canal therapy, supernumerary tooth, tooth abnormalities

## Abstract

**Key Clinical Message:**

Management of supernumerary teeth fused to the labial surface of permanent maxillary central incisors would require a multidisciplinary approach comprising of endodontic treatment, periodontal recontouring, and cosmetic composite restoration.

**Abstract:**

The reported cases of supernumerary teeth fused to the labial surface of maxillary central incisors are rare. Such cases need multidisciplinary approaches. Herein, management of a supernumerary tooth fused to the labial surface of a maxillary central incisor is reported. Due to the presence of a communication path between the root canal systems of the two fused teeth, root canal therapy was performed first for the maxillary left central incisor and the supernumerary tooth. The crown of the supernumerary tooth was then removed in a surgical setting while preserving the root to maintain the thin covering of alveolar bone and prevent future periodontal problems. Subsequently, an esthetic composite restoration was performed.

## Graphical abstract

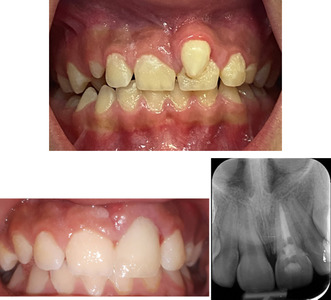

## 1 INTRODUCTION

Fusion is a developmental anomaly of the dental hard tissue, in which the merging of two developing tooth buds results in the formation of a single large dental mass.^1^ The pathogenesis of tooth fusion involves the union of two normally separated tooth germs resulting in a bifid crown and two root canals.^2^ This entity can be complete or partial, depending on the developmental stage in which it occurs.^3^ Tooth fusion can be classified into four types based on the morphology of the fused teeth^4^:

Type 1: bifid crown, single root.

Type 2: large crown, large root.

Type 3: two fused crowns, double conical root.

Type 4: two fused crowns, two fused roots.

Clinically, the fused tooth usually has a wide crown and two independent root canals, or, less commonly, one single root and one or two pulp chambers.^5^ The etiology of fusion has not been fully understood. According to some researchers, this phenomenon results from physical forces exerted on the developing tooth germ, leading to necrosis of the epithelial tissue between the two fused buds. As a consequence, the neighboring buds come into contact and fuse to develop a fused dental structure.^6^, ^7^ Some others believe that fusion arises from the persistence of the interdental lamina between the two buds during embryological development.^8^ Other causes include genetics, racial predisposition, or even trauma during tooth development.^9^, ^10^ Based on the studies of case reports, fusion can be seen in Robinow syndrome^11^ and some rare diseases such as congenital erythropoietic porphyria, hypophosphatasia, Marfan syndrome, and Peutz–Jeghers syndrome.^12^ Fusion can involve permanent, primary, or supernumerary dentition. Permanent maxillary central incisors are among the most commonly affected teeth. According to the literature, the prevalence of fusion ranges from 0.2% to 2.5% among different ethnicities and is more common in the primary dentition.^13^, ^14^, ^15^ Fusion is sometimes mistaken for gemination, which is defined as the attempted formation of two teeth from one single enamel organ.^1^ The incidence of gemination is reported to be 0.47%, with no sex predilection.^16^ Supernumerary teeth in dentition most probably result from continued proliferation of the permanent or primary dental lamina to form a third tooth germ.^17^ Mesiodens is the most common supernumerary tooth which is located between the maxillary central incisors. It is usually in the form of a cone‐shaped crown with a short root. Supernumerary teeth have a prevalence of 0.1%–3.8% in permanent dentition and 0.35%–0.6% in primary dentition, with a predilection for males (2:1).^18^

Several recommended treatments for fused teeth have been discussed in the endodontic^19^, ^20^, ^21^ and orthodontic^22^ literature. In most cases, fusion requires a multidisciplinary approach.^23^, ^24^, ^25^, ^26^, ^27^ Supernumerary teeth fused to the labial surface of maxillary central incisors have been rarely reported in the literature.^28^, ^29^ Herein, management of a supernumerary tooth fused to the labial surface of a maxillary central incisor is reported.

## 2 CASE REPORT

**FIGURE 1:**
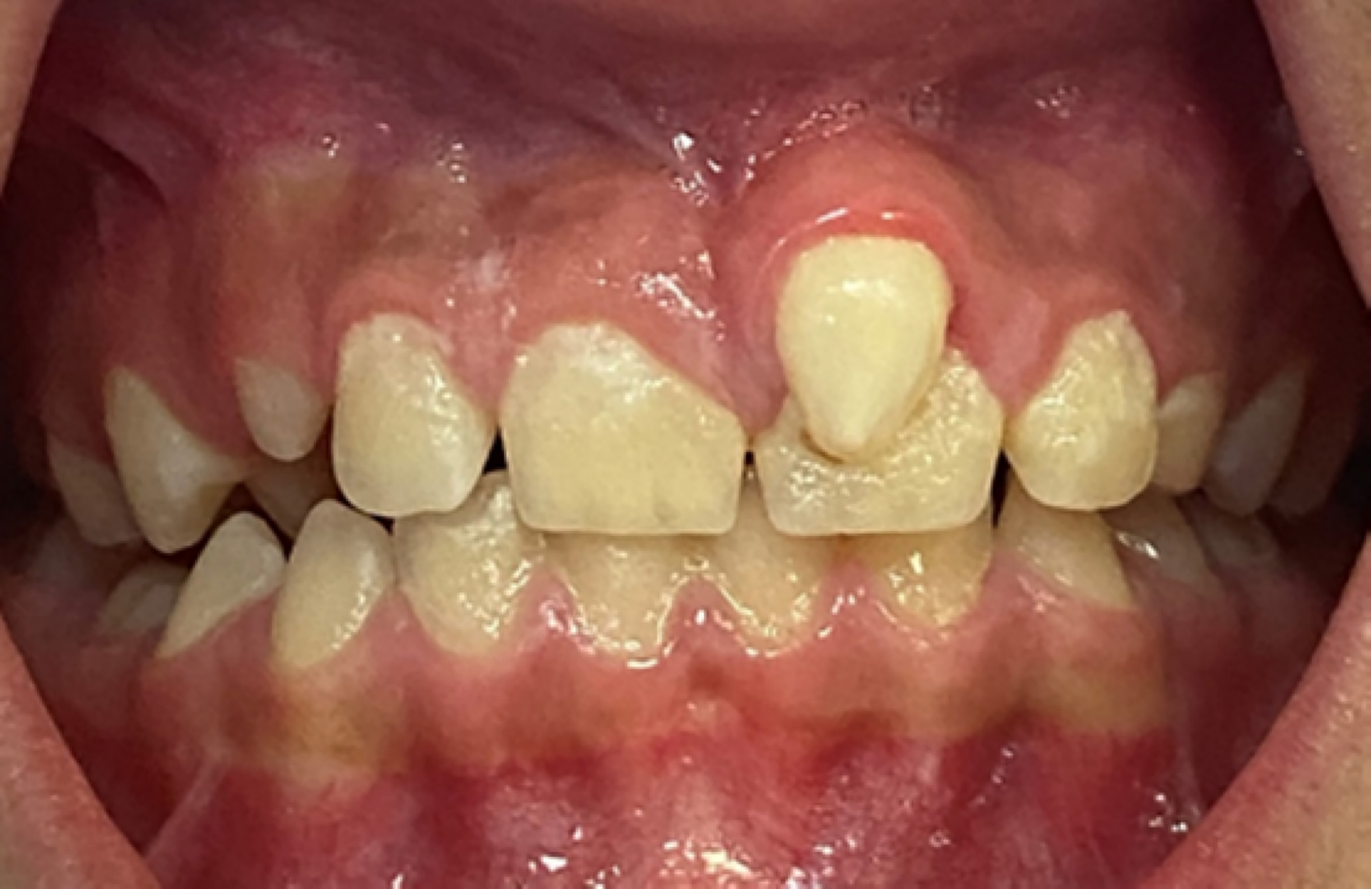
Intraoral photograph showing a conical supernumerary tooth on the labial surface of the maxillary left central incisor.

**FIGURE 2:**
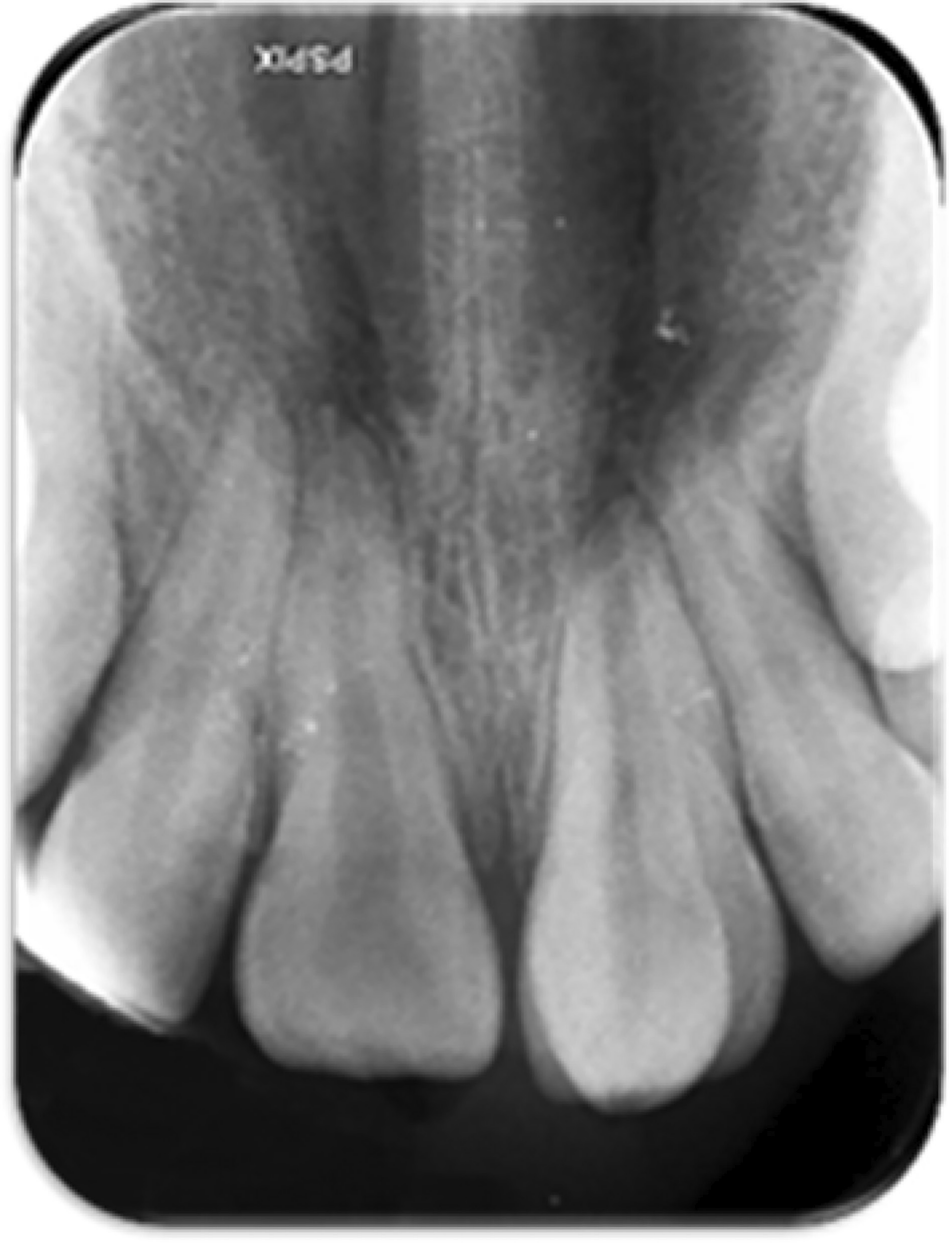
Preoperative periapical radiograph.

**FIGURE 3:**
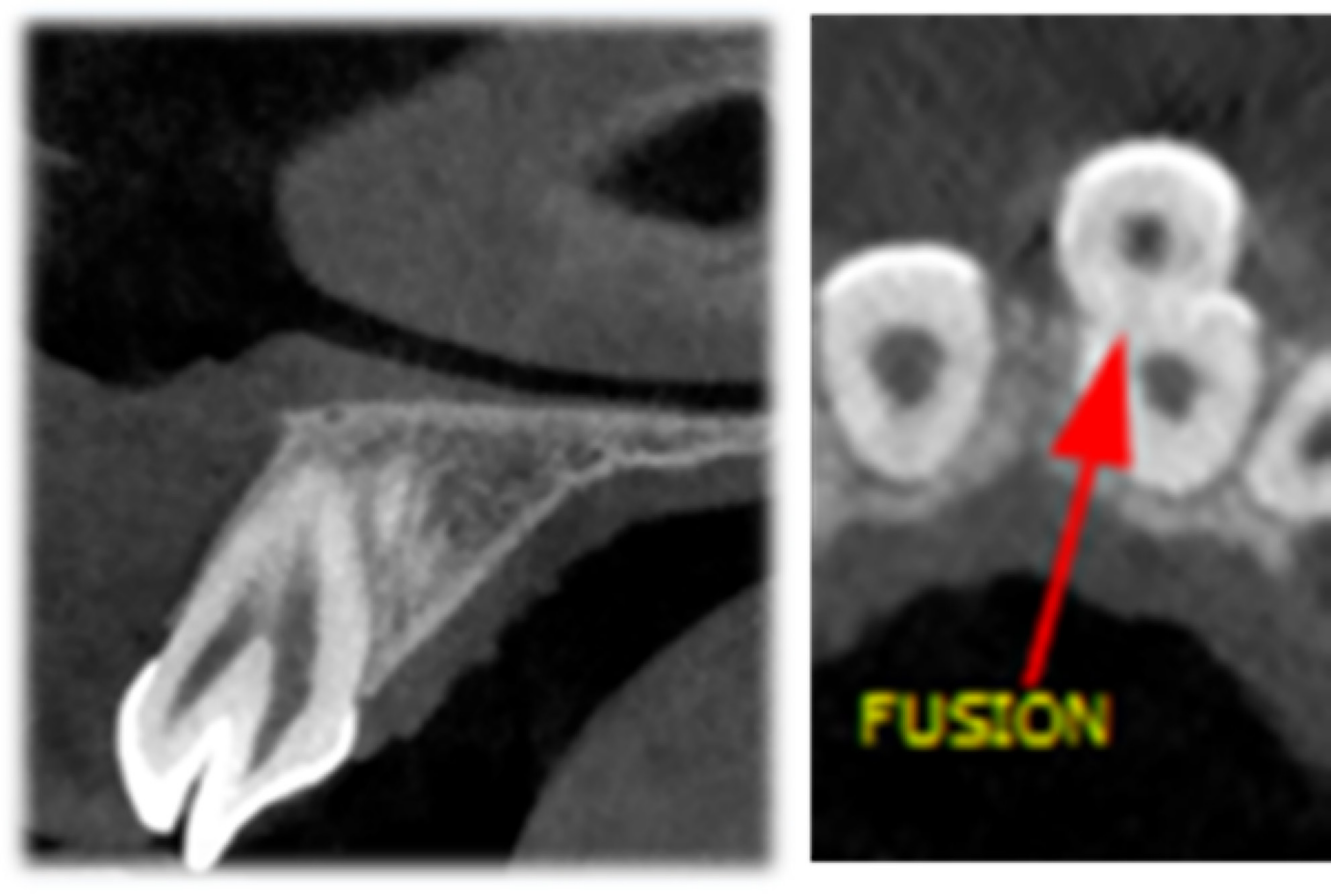
Sagittal and axial cone‐beam computed tomography views of the involved area with a limited field of view.

**FIGURE 4:**
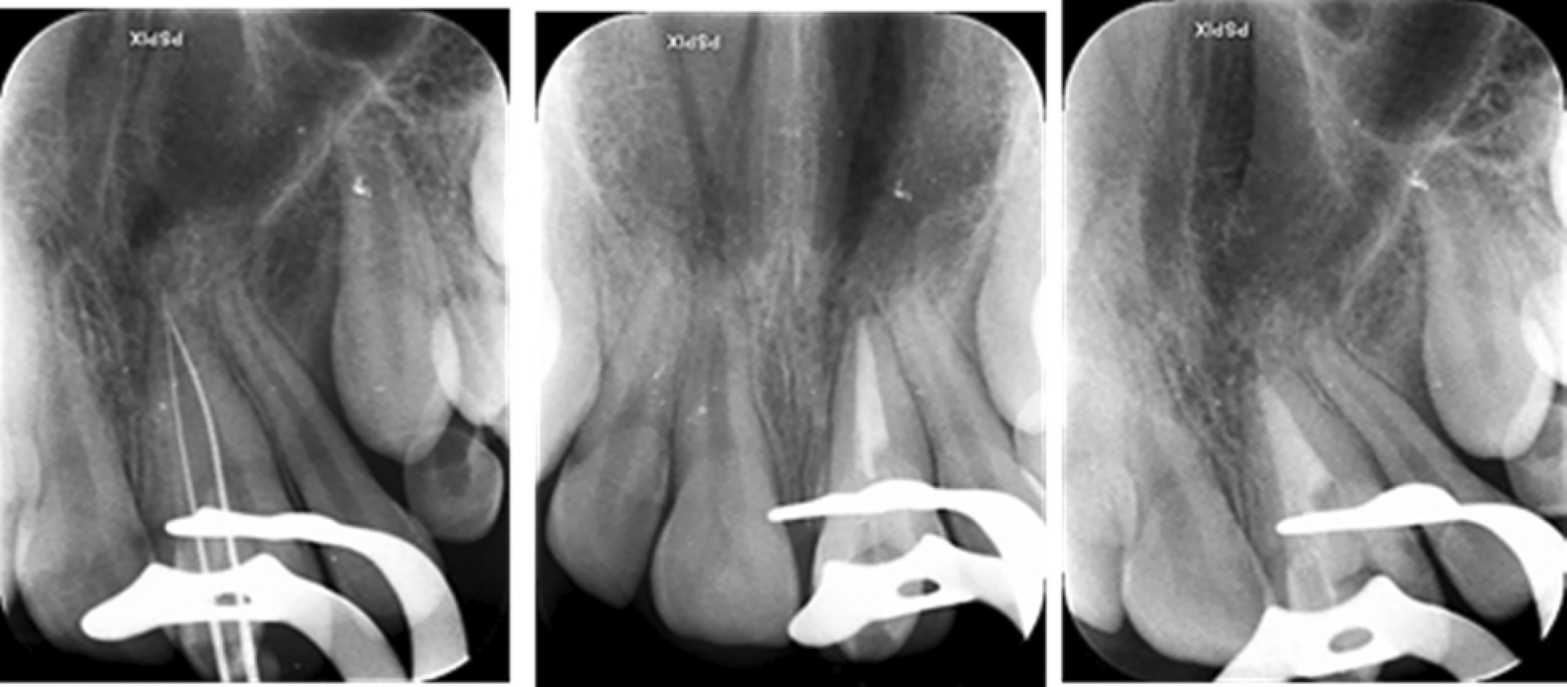
Intraoperative periapical radiographs.

An 11‐year‐old female was referred to the Department of Pediatric Dentistry and Endodontics, School of Dentistry, with a chief complaint of “esthetic problems” on September 12, 2020. The patient had a non‐contributory medical history with no accompanying physical or mental abnormalities. She did not report any dental treatment in the affected area. No previous history of traumatic injury to the jaws or teeth was reported in the affected area. Intraoral examination revealed a conical supernumerary tooth in close contact with the labial surface of the maxillary left central incisor (Figure 1). There was no sensitivity to percussion or palpation in the supernumerary tooth or the central incisor. Both teeth had normal mobility and probing depth and responded positively to the pulp tests. Radiographic examination showed mature apices of the maxillary incisors and a supernumerary tooth fused to the left central incisor (Figure 2). Cone‐beam computed tomography was requested for a more detailed view of the complex root canal system morphology (Figure 3), which revealed a pulpal communication path between the fused and the supernumerary teeth. Therefore, it was decided to perform root canal treatment for both dental structures before surgical recontouring of the involved area. The root canal systems were obturated by Retro‐MTA to provide an adequate seal (bioMTA) up to the coronal communication area and 2 mm apical to the cementoenamel junction (Figure 4).

**FIGURE 5:**
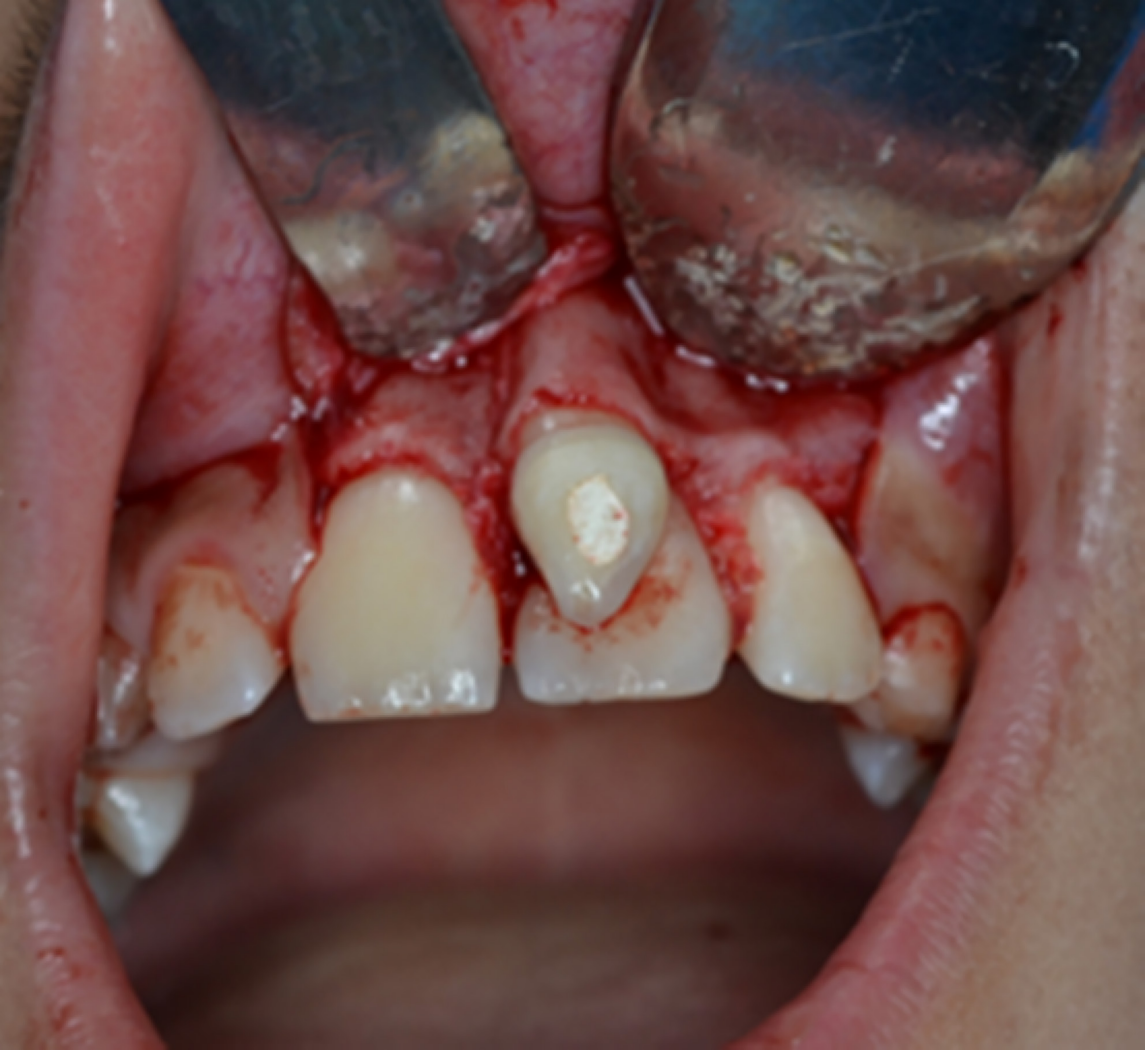
Intraoral photograph after periodontal flap elevation.

**FIGURE 6:**
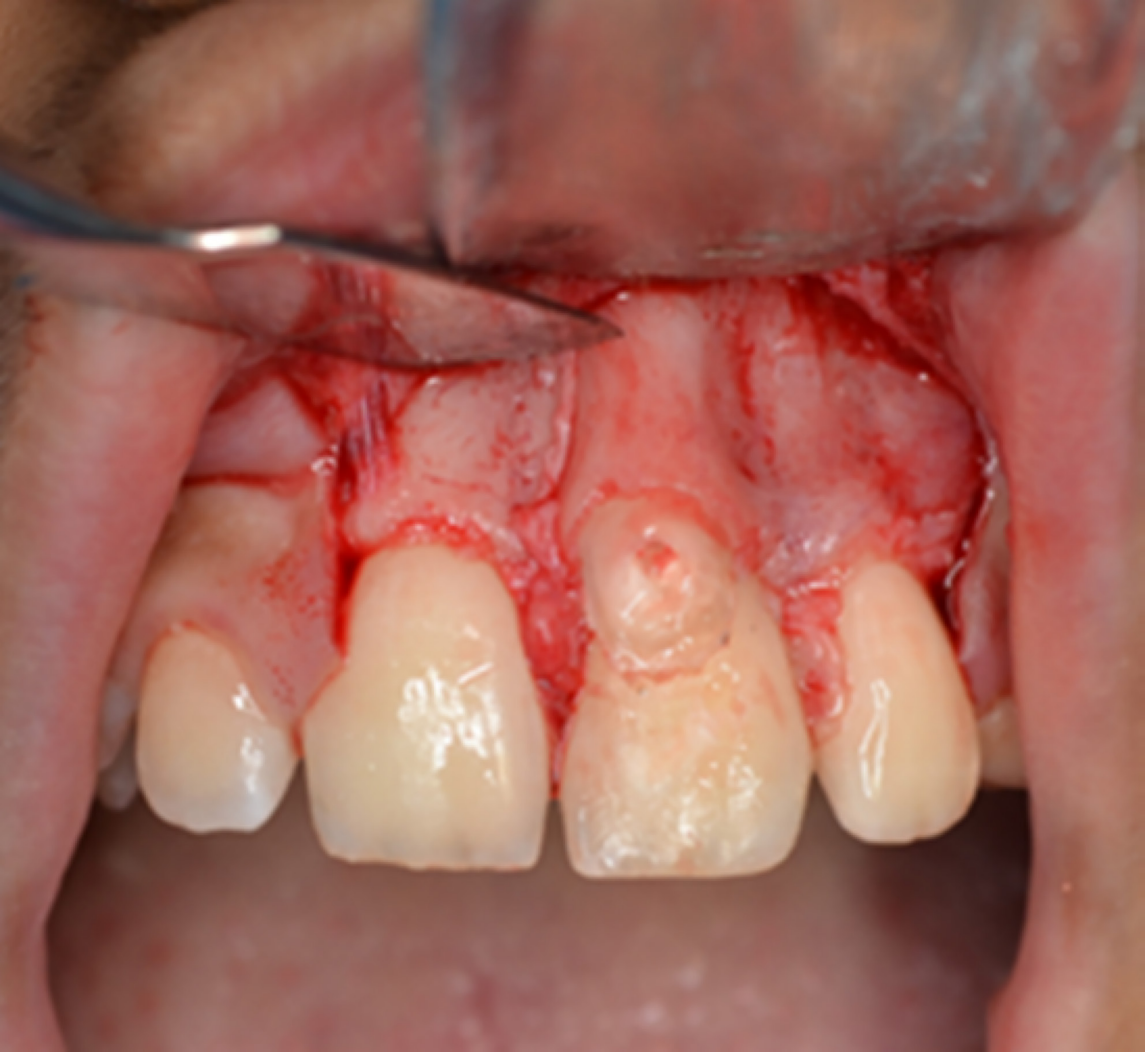
Crown removal of the supernumerary tooth.

**FIGURE 7:**
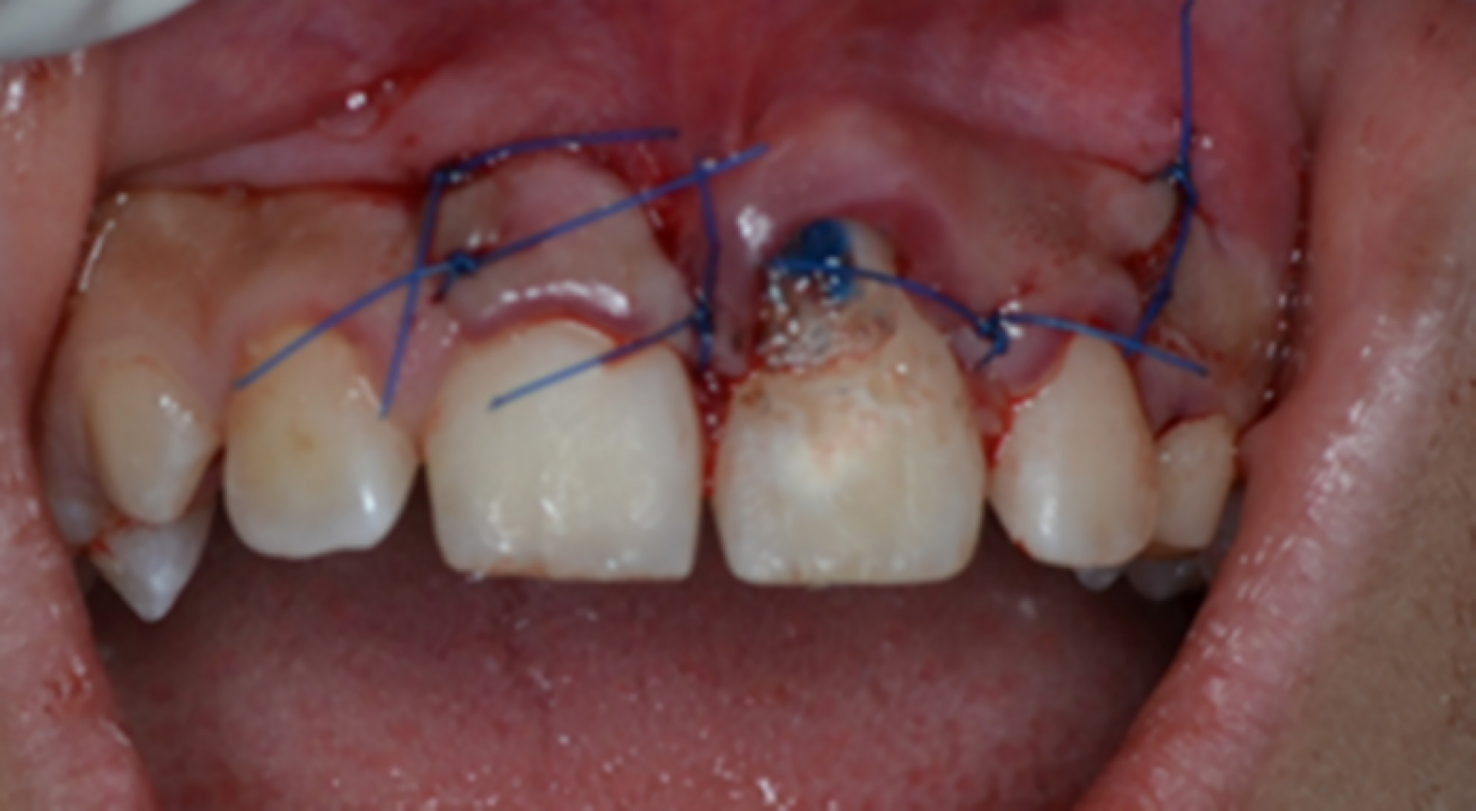
Sealing of the exposed area with light‐cure glass ionomer.

After the completion of endodontic treatment, periodontal surgery was performed to remove the supernumerary tooth and reshape the involved region. A full‐thickness mucoperiosteal flap was elevated, and the surgical area was exposed. Since there was a thin layer of bone covering the radicular portion of the supernumerary tooth (Figure 5), only the crown of the supernumerary tooth was removed in a beveled form to preserve the remaining osseous layer (Figure 6). Then, the empty pulpal space of the supernumerary tooth coronal to the fused area was sealed with light‐cure glass ionomer cement (FUJI II LC Improved, GC). Eventually, the flap was repositioned and sutured in place (Figure 7). A recall visit was scheduled 10 days after the procedure, and the sutures were removed. The access cavity was etched with 37% phosphoric acid (Morvabon) for 20 s. Then, a dentin bonding agent (Single Bond, 3M, ESPE) was applied and light‐cured. Afterwards, the cavity was permanently restored with composite resin (TECO, P.L. Superior Dental Materials). The labial surface of the tooth received an esthetic restoration using the abovementioned restorative materials.

**FIGURE 8:**
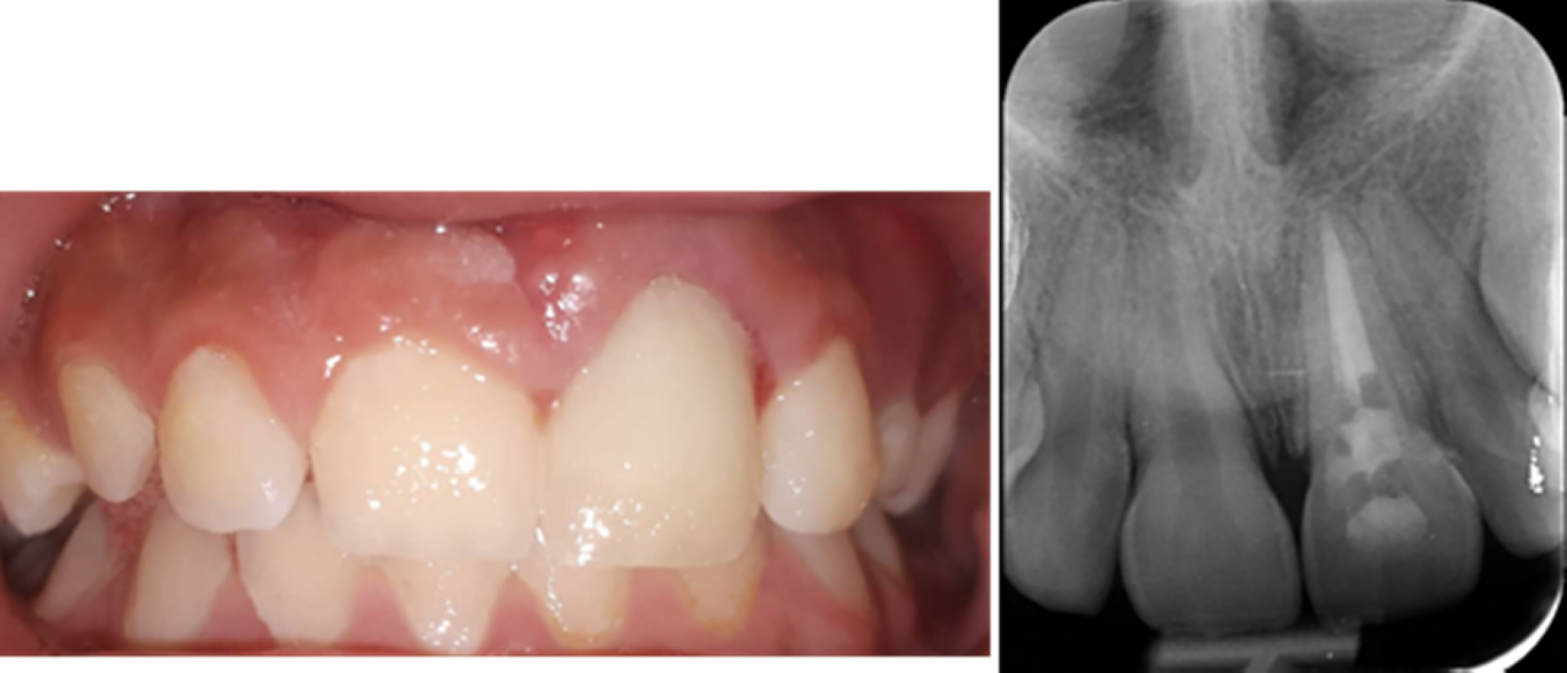
Intraoral photograph and periapical radiograph taken at the 6‐month follow‐up.

At the 6‐month follow‐up, the periradicular tissues appeared normal. The probing depth was within the normal range, and the patient was satisfied with the esthetic outcome (Figure 8).

## 3 DISCUSSION

Fusion is a rare dental anomaly that may occur in primary, permanent, or even supernumerary teeth. Its diagnosis can be challenging since it may be mistaken with gemination when it coincides with hypodontia or hyperdontia. Fusion of a permanent and a supernumerary tooth is an infrequent occurrence compared to fusion of primary teeth. The incidence of fusion of a permanent and a supernumerary tooth is reported to be 0.1%, and it generally involves the maxillary anterior teeth.^30^ Although the fusion of two dental structures frequently takes place in proximal areas, the supernumerary tooth had fused to the buccal surface of the maxillary left central incisor in the present case.

The best treatment plan depends on the nature of the fusion, the age of the patient, and the patient's overall dental health. Here are some of the interdisciplinary treatment methods that have been proposed; Orthodontic treatment can be used to correct the alignment of fused teeth and improve the patient's bite. This treatment may involve the use of braces, aligners, or other orthodontic appliances.^24^, ^31^ EEndodontic treatment may be necessary if the pulp of the fused teeth is damaged or infected. This treatment involves removing the damaged pulp and filling the root canals with a special material.^24^, ^32^ Restorative treatment can be used to repair the appearance and function of fused teeth. This treatment may involve the use of dental crowns, veneers, or other restorative materials.^24^, ^31^ In some cases, surgical treatment may be necessary to separate fused teeth. This treatment may involve the use of a dental drill or other surgical instruments to separate the teeth.^24^, ^32^ Various multidisciplinary approaches have been suggested in the literature for the management of fused incisors.^23^, ^24^, ^25^, ^26^, ^27^ In the current case, orthodontic and prosthodontic interventions were not indicated because of the labial position of the fused supernumerary tooth. However, the development of future periodontal problems was probable in the area. Thus, endodontic treatment was performed for both the supernumerary tooth and the maxillary left central incisor because of the presence of a pulpal connection path between the fused teeth. Simultaneous endodontic treatment of the fused and supernumerary teeth was also performed according to Oelgiesser et al.^33^ and Brunet‐Llobet et al.^31^ due to the presence of a pulpal communication path between the two root canals.

Several materials have been proposed for root canal obturation, including calcium silicate cements, gutta‐percha, and different types of sealers.^34^, ^35^ Regarding the material used for better root treatment of dental fusions, there is no specific material mentioned in the search results. However, some studies have reported successful endodontic therapy and direct resin composite restoration for fused teeth with separated root canals. In this case, the root canal system was obturated with RetroMTA (bioMTA) due to the possibility of exposure of the root canal filling material during surgical removal of the supernumerary tooth and reshaping of the fused area. On the other hand, since there was no need for post‐space preparation for the restorative procedure due to the presence of an adequate bondable coronal structure, RetroMTA appeared to be a suitable root canal filling material. RetroMTA is a fast‐setting calcium silicate endodontic cement with an initial setting time of about 180 s. The main chemical components of RetroMTA include calcium carbonate, silicon dioxide, aluminum oxide, and the calcium zirconia complex.^36^, ^37^ Tooth discoloration following the application of MTA is a major esthetic concern in the anterior region.^38^ In this case, RetroMTA was used as the root canal filling material, and the root canal space was obturated 2 mm apical to the cementoenamel junction. According to a systematic review of in vitro studies, some calcium silicate‐based cements such as gray and white MTA Angelus, gray and white ProRoot MTA (Dentsply), and Ortho MTA (BioMTA) have high potential to cause tooth discoloration. However, Biodentine (Septodont), RetroMTA (BioMTA), Portland cement, EndoSequence Root Repair Material (Brasseler USA), Odontocem (Australian Dental Manufacturing), MM‐MTA (Micro Mega, Besancon Cedex), and MTA Ledermix (Riemser Pharma GmbH) were reported to have the lowest staining potential.^39^

Additionally, the coronal extension of the root canal filling material was set 2 mm apical to the cementoenamel junction to avoid discoloration due to penetration of root filling particles into the dentinal tubules in the cervical region of the access cavity.

Another major concern while using MTA is its washout potential following exposure to fluid flow.^40^ In the current case, the exposed area communicated with the oral cavity through the gingival sulcus after surgical removal of the supernumerary tooth. Therefore, the exposed area was covered with a thin layer of glass ionomer cement as a protector^41^ to avoid washout.

## AUTHOR CONTRIBUTIONS

**Hadi Assadian:** Investigation; methodology; writing – review and editing. **Behnam Bolhari:** Investigation; methodology. **Mehrfam Khoshkhounejad:** Investigation; supervision; writing – original draft. **Nazanin Chitsaz:** Supervision; validation. **Maryam Babaahmadi:** Methodology; writing – original draft.

## FUNDING INFORMATION

None.

## CONFLICT OF INTEREST STATEMENT

The authors deny any conflict of interest.

## ETHICS STATEMENT

For clinical cases, the local ethics committee considers that the patient's consent is sufficient.

## CONSENT

Written informed consent was obtained from the patient to publish this report in accordance with the journal's patient consent policy.

## Data Availability

The data supporting the findings of the present study, are available from corresponding author upon request.
